# Serum-Free Culture of Human Mesenchymal Stem Cell Aggregates in Suspension Bioreactors for Tissue Engineering Applications

**DOI:** 10.1155/2019/4607461

**Published:** 2019-11-07

**Authors:** Leah M. Allen, John Matyas, Mark Ungrin, David A. Hart, Arindom Sen

**Affiliations:** ^1^Pharmaceutical Production Research Facility, Department of Chemical and Petroleum Engineering, Schulich School of Engineering, University of Calgary, Calgary, AB, Canada; ^2^Biomedical Engineering Graduate Program, University of Calgary, Calgary, AB, Canada; ^3^McCaig Institute for Bone and Joint Health, Cumming School of Medicine, University of Calgary, Calgary, AB, Canada; ^4^Department of Comparative Biology and Experimental Medicine, Faculty of Veterinary Medicine, University of Calgary, Canada; ^5^Alberta Children's Hospital Research Institute, University of Calgary, Calgary, AB, Canada; ^6^Faculty of Kinesiology, University of Calgary, Calgary, AB, Canada; ^7^Alberta Health Services Bone & Joint Health Strategic Clinical Networks, AB, Canada

## Abstract

Mesenchymal stem cells (MSCs) have the capacity to differentiate towards bone, fat, and cartilage lineages. The most widely used culture and differentiation protocols for MSCs are currently limited by their use of serum-containing media and small-scale static culture vessels. Suspension bioreactors have multiple advantages over static culture vessels (e.g., scalability, control, and mechanical forces). This study sought to compare the formation and culture of 3D aggregates of human synovial fluid MSCs within suspension bioreactors and static microwell plates. It also sought to elucidate the benefits of these techniques in terms of productivity, cell number, and ability to generate aggregates containing extracellular matrix deposition. MSCs in serum-free medium were either (1) inoculated as single cells into suspension bioreactors, (2) aggregated using static microwell plates prior to being inoculated in the bioreactor environment, or (3) aggregated using microwell plates and kept in the static environment. Preformed aggregates that were size-controlled at inoculation had a greater tendency to form large, irregular super aggregates after a few days of suspension culture. The single MSCs inoculated into suspension bioreactors formed a more uniform population of smaller aggregates after a definite culture period of 8 days. Both techniques showed initial deposition of extracellular matrix within the aggregates. When the relationship between aggregate size and ECM deposition was investigated in static culture, midsized aggregates (100-300 cells/aggregate) were found to most consistently maximize sGAG and collagen productivity. Thus, this study presents a 3D tissue culture method, which avoids the clinical drawbacks of serum-containing medium that can easily be scaled for tissue culture applications.

## 1. Introduction

Mesenchymal stem cells (MSCs) have the capacity to self-renew and differentiate towards multiple lineages including bone, cartilage, fat, muscle, and tendon, if subjected to the appropriate respective culture conditions [1, 2]. They can also be isolated from various adult tissue sources including bone marrow, adipose tissue, synovial membrane, synovial fluid, and periosteum [2–6]. Furthermore, MSCs have unique therapeutic characteristics, which include homing to tissue injury sites, the ability to produce large quantities of trophic factors, and exerting immunomodulatory effects [7]. Due to the therapeutic characteristics observed with these cells, they are currently being evaluated in over 900 registered clinical trials (https://ClinicalTrials.gov).

MSCs have traditionally been expanded in serum-containing media. However, this is problematic because serum is ill-defined, and there are often large variations between batches of serum, creating inconsistencies in experimental results and making standardization of bioprocesses challenging [8]. Furthermore, animal-derived sera may contain prion, viral, and zoonotic agents which can lead to immune reactions [9]. To overcome these challenges to the clinical translation of MSC-based technologies, defined serum-free media have been developed for the culture of MSCs. PPRF-msc6 is one such medium that has been shown to be effective for the rapid and reproducible isolation and expansion of human MSC populations derived from the bone marrow [9, 10].

MSCs have traditionally been cultured as a monolayer on a two-dimensional (2D) surface in static tissue culture flasks. However, expanding cell populations in this manner is labour intensive and not conducive to reproducibly generating the large numbers of cells required for tissue-engineering applications. Whereas attempts to expand MSCs as three-dimenstional (3D) aggregates in static culture have been met with very limited success [11], expansion of MSCs in 3D aggregates in suspension culture has been reported to support expansion similar to that observed in 2D culture [12, 13]. 2D culture flasks have not traditionally been used to facilitate the induction of MSCs into 3D forms, which may be superior to 2D culture systems because of enhanced MSC differentiation, tissue development, and therapeutic benefits that are thought to be mediated through cell-cell contact and cell-extracellular matrix (ECM) interactions [12, 14, 15].

Due to ease of research and cost considerations, 3D-engineered tissues are often produced within small-scale vessels (i.e., centrifuge tubes and standard well plates) using a variety of formation techniques (e.g., cell seeding on biomaterials, pellet culture, micromass, and hanging drop methods) [14, 16–19]. However, these aforementioned culture methods are either manually intensive, not amenable to scaled-up production, cost-prohibitive from a therapeutic perspective, and/or cannot be subjected to mechanical stimulation. More recently, a new method has been described in which MSCs are seeded at very high density on 2D static well plates and induced to secrete high levels of extracellular matrix molecules [20–22]. After 3-21 days, the layer of cells and ECM are released from the plate and proceed to spontaneously contract into a 3D tissue-like structure. This new method of 3D tissue formation has proven to be reproducible and to have good incorporation and integration into native human cartilage defects [23]. This approach is amenable for the production of variable sizes of tissue to fill cartilage defects, as such scale-up is not an issue. However, mechanical stimulation is not inherent to this formation technique.

The mechanical environment, including shear and hydrostatic pressure, is an important consideration for a variety of tissues. For example, *in vivo*, articular cartilage is subjected to mechanical stimulation through the normal loading cycles of the joint. Cells are able to sense mechanical stimulation through mechanotransduction, which converts mechanical stimuli into biochemical signals that elicit a cellular response, such as changes in gene regulation and ECM production [24]. Efficient approaches for tissue formation that incorporate mechanical stimulation may serve to generate better cell constructs for cartilage repair.

Suspension bioreactors have been used to culture a number of different stem cell types [25–27], including MSCs [12, 13, 28–30], and are not subjected to the same scaling limitations as conventional culture methods. Other benefits include ease of handling, enhanced control and homogeneity of the culture environment, and exposure to mechanical forces such as fluid shear stress [31]. Flow within suspension culture is known to demonstrate a Reynolds number in the upper transition zone between laminar and turbulent [32]. The energy from the impeller also produces eddies that form pockets of alternate mixing. Within the bioreactor, the aggregates are not confined to one area. As a consequence, each individual aggregate should see the same amount of volume average shear, calculated to be 11.5 s^−1^ based on previously published models [33].

Shear is a prominent mechanical stimulus within a stirred suspension bioreactor, induced by the fluid flow created by the impeller rotation. Mechanical shear on chondrocytes has been shown to enhance the quality of cartilaginous tissue formed in vivo [34]. Due to the aforementioned benefits, suspension bioreactors may prove to be the superior method in culturing tissue-like structures with regenerative properties from MSCs. Formation and culture of the MSC aggregates in the suspension bioreactors can vary; they can either be aggregated together within the bioreactors [13] or be aggregated together in a static environment and then inoculated into the bioreactors.

Microwell plates have emerged as a potential high-throughput method for creating uniform populations of MSC aggregates (spheroids) [14, 35–37]. Compared to other 3D formation techniques, the formation of aggregates using this system is simple. A single cell suspension is distributed over a surface containing thousands of microwells, and when centrifuged, tens to thousands (depending on suspension density) of cells are deposited into each microwell. Subsequent incubation allows the cells to adhere to one another and form a spherical aggregate of controlled size and composition [38]. This study is aimed at culturing aggregates of MSCs within the dynamic environment of stirred suspension bioreactors using two methods: (1) single cell inoculation into suspension bioreactors at a density high enough to encourage spontaneous cell aggregation or (2) preformation of aggregates within microwell plates, followed by inoculation of these aggregates into suspension bioreactors for culture. Aggregates formed and cultured within static microwell plates were also included in this study for comparison. After culture, we sought to characterize the size, uniformity, and subsequent chondrogenic quality of the MSC aggregates. Priming cells to differentiate towards a chondrogenic lineage prior to implantation may make them more effective candidates for the repair of cartilage defects, as the local chemical and mechanical signals *in vivo* could better promote the formation of durable cartilage tissue [23].

## 2. Methods and Materials

### 2.1. Human Synovial Fluid MSC Isolation and Expansion

Nonosteoarthritic cadaveric human synovial fluid was obtained through the tissue donation program at the University of Calgary (protocol #REB150005). Synovial fluid (200 *μ*L) was added to 2.0 mL of warmed PPRF-msc6 medium, a serum-free medium formulated for the growth of MSCs [9, 39], and then dispensed into a 6-well plate (VWR, Cat. No. 10861-554) coated with gelatin (0.1% solution) (Sigma-Aldrich, Cat. No. G6650). The plates were placed in a humidified incubator at 37°C and 5% CO_2_. Upon reaching sufficient confluency (70-90% total surface area covered with cells), the adherent cells were released from the plate by incubating the cells in TrypLE Express (Life Technologies, Cat. No. 12605-028) in a humidified incubator at 37°C for 3-4 minutes. After detachment, they were transferred to a sterile conical tube, pelleted by centrifugation, resuspended in fresh PPRF-msc6 medium [10, 39], and placed into gelatin-coated flasks at a seeding density of 5000 cells/cm^2^. Passage 2 cells were assessed for their expression of reported MSC cell surface markers (i.e., positive expression of CD90, CD105, and CD73 and negative expression of CD14, CD34, and CD45) by flow cytometry [3]. To reach the number of cells needed to inoculate the suspension bioreactors and microwell plates, the cells were serially passaged by the same method previously described. Passage 5 cells were used in all experiments. Previous studies have shown that MSCs maintained their proliferative and morphological characteristics over at least 10 passages in the serum-free medium that was used in these experiments [39].

### 2.2. Microwell Plate Experiments

MSCs grown as adherent monolayers on static culture plates were passaged then aggregated together in static microwell plates (AggreWell 400, STEMCELL Technologies Inc., Vancouver). Microwell plates were prepared as previously reported [35]. MSCs in suspension after passaging were inoculated at the desired cell density per microwell using 800 *μ*L of PPRF-msc6 medium in a 24-well microplate. Within the microwell plates, spheroids that formed during overnight incubation at 37°C through cell contraction, and that did not break apart during subsequent gentle pipetting, constituted an aggregate. Aggregate formation was verified using an inverted light microscope.

### 2.3. Bioreactor Inoculation and Culture

Suspension bioreactors (125 mL) with paddle impellers (NDS Technologies, Cat. No. 264501-125) were used for the suspension culture experiments. The suspension bioreactors were siliconized to prevent cell attachment by coating the vessel and impellor with 10 mL of Sigmacote (Sigma-Aldrich, Cat. No. SL-2), then conducting serial washings of PBS and ddH_2_O prior to sterilization using an autoclave. In accordance with our previous work with MSCs in suspension culture [40, 41], a working volume of 125 mL was used in the bioreactors and the cells were inoculated at 50,000 cell/mL in PPRF-msc6 medium. Single cells were inoculated into a suspension bioreactor on culture day 0 after being harvested from culture flasks. Based on standard methods developed in our laboratory [29], the suspension bioreactors were placed on a magnetic stir plate set at 80 rpm inside a humidified 5% CO_2_ incubator at 37°C. A 50% medium change was carried out every fourth day throughout the culture period by halting impeller motion, allowing the aggregates to settle briefly and carefully drawing off half of the spent medium.

The other test condition evaluated was the inoculation of preformed aggregates (as compared to the single cell condition described above) into the suspension bioreactors. To preform the aggregates, cells were inoculated into microwell plates at a density of 500 cells/microwell. Aggregates that formed overnight within the microwell plates were harvested and inoculated into a suspension bioreactor on culture day 1. As such, the start of the aggregation from single cells after passage 5 either in the suspension bioreactors or the microwell plates was held constant and denoted as culture day 1. The suspension bioreactors inoculated with the preformed aggregates were cultured using our standard protocol as described above.

To serve as a static control, aggregates were formed in microwell plates and kept in the individual microwells over the culture period. Due to the high density of cells within the plates for this condition, a 50% medium change was conducted on the plates daily. To perform the medium change, spent medium was carefully drawn off in 100 *μ*L increments from the side of the well plate and gently replaced with an equal quantity of fresh medium in 100 *μ*L increments.

### 2.4. Aggregate Imaging, Sizing, and Cell Viability

To visualize the aggregates and obtain measurements of their diameters, 1.0 mL samples were taken every other day from each of the replicate suspension bioreactors and transferred to a 24-well plate (VWR, Cat. No. 10861-558). Photomicrographs were taken using a Leica DMIL 351 microscope (Leica Microsystems GmbH, Wetzlar, Germany) equipped with a QIMAGING digital camera (QIMAGING, Canada) and OpenLab 5.5 Image Processing software (Improvision, Waltham, MA). For the preformed aggregates kept in static culture, a representative 10% of the aggregates from each replicate microwell plate (approximately 120 aggregates) were transferred to a 24-well plate for aggregate sizing. In the single-cell bioreactor condition, aggregates of less than 40 *μ*m in diameter were not considered in the calculations, as they were generally single cells or doublets that were too small to accurately measure (Supplementary [Supplementary-material supplementary-material-1]). Aggregates formed using this method were found to remain intact.

### 2.5. Macromolecule Quantification

Once the cells were formed into aggregates, they progressively produced ECM throughout the culture period and were then deemed tissue-like aggregates (aggregates of MSCs that upregulate production of ECM). Quantification of sGAG and DNA was conducted on samples of tissue-like aggregates harvested from the bioreactors and the static microwell plates. Duplicate samples of tissue were harvested from three replicate bioreactors, washed in 1x PBS, and then digested overnight at 56°C in a Proteinase K working solution containing 1 mg/mL Proteinase K (Sigma, Cat. No. P2308), 50 mM EDTA, 1 mM iodoacetamide, and 10 *μ*g/mL Pepstatin A (Sigma, Cat. No. P5318). Measurement of DNA content was performed with a CyQUANT Cell Proliferation Assay Kit (Life Technologies, Cat. No. C7026) according to the manufacturer's protocol. Using a fluorescence plate reader, the samples were excited at a wavelength of 485 nm and the emission read at a wavelength of 525 nm. All plate readings were performed in triplicate. Fluorescence values were correlated to DNA concentration using a standard curve generated using known quantities of bovine DNA. Proteinase K working solution containing no tissue/cells was used as the normalizing control. DNA mass was converted into cell numbers using the conversion of 6.6 pg DNA/diploid primary cell [42]. This conversion was calculated through knowledge that MSCs are diploid, that there are roughly 3 billion base pairs per haploid set of DNA, the molecular weight of each base pair, and the mass of each base pair in daltons (3 × 10^9^ bp × 2 (diploid) × 660 (MW of 1 bp) × 1.67 × 10^−12^ pg = 6.6 pg/diploid primary cell).

The quantity of sGAG in the proteinase K/tissue digest was measured by 1,9 dimethyl methylene blue zinc chloride double salt (DMMB) (Sigma-Aldrich, Cat. No. 341088) reaction. The colorimetric changes were measured using a plate reader at 525 nm. Absorbance values were translated back to sGAG concentration using a standard curve generated with known quantities of chondroitin sulphate (Supplementary [Supplementary-material supplementary-material-1]). All experimental samples from the bioreactors and the microwell plates ranged from approximately 11 to 63 *μ*g sGAG per mL of proteinase K working solution, which were all within the limits of the standard curve. Proteinase K working solution containing no aggregate tissue was analyzed as the normalizing control. sGAG was further normalized to DNA to allow for comparison between experimental conditions and with cited values for normal articular cartilage and other engineered cartilage tissue [37, 43–45].

### 2.6. Macromolecule Staining: Histology and Immunohistochemistry

Histology was performed on the tissue-like aggregates generated in suspension culture to detect and visualize GAGs, collagen type I, and collagen type II. The tissue was washed in 1x PBS and then fixed in 4% paraformaldehyde. The samples were processed through a series of alcohol gradients for dehydration, embedded in paraffin, and cut into 8 *μ*m sections, which were adhered onto glass slides.

To detect GAG deposition, the tissue sections were deparaffinized and rehydrated using 70% ethanol for 2 minutes, then quickly dipped ten times in each of 95% and 100% ethanol. They were then stained with 1 mg/mL toluidine blue in 0.9% NaCl solution (pH 2.3) for 3 min. The slides were dehydrated quickly using 70% ethanol for 2 min, dipped alternately into 95% and 100% ethanol for 10 cycles each, and transferred through two changes of xylene for 3 minutes each before being covered with a coverslip.

Immunohistochemistry (IHC) was employed to detect collagen type I and type II within the MSC aggregates. Briefly, sectioned tissue was rehydrated through a series of ethanol gradients and then subjected to 0.01% hyaluronidase antigen retrieval. Endogenous peroxidase activity was then blocked using 0.3% H_2_O_2_ in 90% methanol. To block unspecific staining of the secondary antibody, the sections were incubated in 10% normal goat serum in 1% BSA/PBS. Half of the sections were incubated with the primary antibodies for collagen type I (Novus Biologicals, 5D8-G9) and collagen type II (DSHB, CCIIC1) in 1.5% normal goat serum in 1x PBS, while the other half were incubated in 1.5% normal goat serum in 1x PBS (negative control). After incubation for 18 hours at 37°C, the secondary antibody (Millipore, Cat. No. 21538) was applied for 1 hour. HRP-streptavidin complex (Invitrogen, Cat. No. 434323) was then applied to the tissue sections and visualized using a 3,3′-diaminobenzidine tetrahydrochloride (DAB) substrate (Invitrogen, Cat. No. 00-2014). Specific positive and negative tissue controls were included to verify the protocol and the reagents used. For collagen type I, human skin was used as the positive control and bovine articular cartilage was used as the negative control. For collagen type II, articular cartilage and subchondral bone from a bovine osteochondral plug were used for both the positive and negative controls, respectively.

Immunostained and toluidine blue-stained tissue sections were imaged using DIC illumination on a Zeiss Axioplan 2 microscope equipped with 5x (NA 0.15), 10x (NA 0.30), and 20x (NA 0.30) objectives. IHC staining was quantified using ImageJ (National Institutes of Health, USA) and an IHC profiler open source plugin that quantifies the pixels stained with DAB [46]. Positive pixels were normalized to the area of aggregate within the image.

### 2.7. Second Harmonic Generation Imaging

Second harmonic imaging (SHG) has proven to be an effective noninvasive 3D method to evaluate collagen deposition and structure within engineered tissues [47]. In an effort to further visualize the collagen deposition within the aggregates and verify the results obtained using the other analyses described, SHG images were collected. To compare the collagen deposition with other ECM and cells contained within the tissue, autofluorescence (AF) was used in conjunction with SHG. Using a Chameleon Ultra fs-pulsed laser source (Coherent Inc.), AF and SHG signals were excited at a wavelength of 780 nm with a pulse width of approximately 140 fs and a repetition rate of 80 MHz. The AF and SHG emission signals were collected using the non-descanned detection (NDD) module (LSM binary GaAsP module, Zeiss) coupled to a Zeiss LSM 710 confocal laser scanning microscope. A 690 nm dichroic mirror attached to the side port of the NDD was used to block the SHG excitation source at 780 nm. The AF and SHG emission wavelengths were separated and collected on the two NDD channels using a filter block, which included two bandpass filters (500-550 nm and 380-402 nm) and a dichroic (495 nm) (Semrock Inc., New York, NY). The SHG signal indicates the collagen deposition within the tissue, whereas the AF signal indicates the entirety of the tissue-like aggregates (cells, sGAG, other ECM, etc.).

Quantification of the SHG signal was completed using ImageJ software (v1.52, NIH, USA) [48]. Background intensity was removed using the Subtract Measured Background macro [49]. The SHG and AF signals for individual aggregates were obtained separately and quantified by outlining the aggregate in their respective stacks.

### 2.8. Oxygenation Considerations

All cell cultures were maintained in a 37°C incubator filled with ambient atmosphere (laboratory elevation approximately 1100 m, air (station) pressure = 0.884 atm) that was humidified and supplemented with 5% CO_2_. Due to equipment limitations, empirically determining oxygen concentration at the surface and within the aggregate under different culture conditions was not possible. Mathematical modelling calculations for both the static microwell plates (medium depth 4 mm; O_2_ consumption rate 27.22 amol/cell · s; cell density 300,000 cell/cm^2^) and the suspension bioreactors were performed using previously reported methods [50]. The oxygen concentration at the cell surface within the microwell plates was 4.32 × 10^−5^ mol/L and the bioreactors was 1.57 × 10^−4^ mol/L (assuming complete mixing). In both conditions the cells on the surface of the aggregates theoretically received adequate oxygen to meet the previously reported oxygen consumption rate of 27.22 amol/cell · s for human mesenchymal stem cells [51]. Previous studies show that MSC aggregates begin to see oxygen diffusion gradients within the core at 60,000 cells per aggregate (approximately 350 *μ*m diameter) [52]. This limitation was taken into consideration for any experimental condition that demonstrated a diameter > 350 *μ*m.

### 2.9. Statistical Analysis

Data are presented as mean ± SD. Statistical analysis was performed using a one-way or two-way ANOVA with Tukey's post hoc test. All statistical analyses were performed in PRISM. v. 7 software (GraphPad, San Diego, CA). Significance has been denoted by alphabetical lettering: groups within which there are no statistically significant differences are linked by the same letter, while groups with statistically significant differences do not share the same letters (*p* < 0.05 being statistically significant). Significance has also been shown by the presence of asterisks between groups (^∗^*p* < 0.05, ^∗∗^*p* < 0.01, ^∗∗∗^*p* < 0.005, and ^∗∗∗∗^*p* < 0.001).

## 3. Results and Discussion

### 3.1. MSC Isolation and Aggregation in Microwell Plates

The human cells used in this study were confirmed to be MSCs through their ability to attach to, and divide upon, culture grade plastic, their spindle-like appearance, their multipotency, and their surface marker expression. Surface marker expression was positive for CD90 (100%), CD105 (99.9%), and CD73 (100%) and negative for CD14 (1.6%), CD34 (1.1%), and CD45 (1.0%), conforming to the definition for MSCs [1, 3, 53]. A chart depicting surface marker expression and antibodies used, as well as phase-contrast light microscopy showing spindle-like shape of the adherent cells, conforming to the morphology of serum-free isolated human MSCs studied previously in our lab [39], is shown in the supplemental text (Supplementary [Supplementary-material supplementary-material-1] & [Supplementary-material supplementary-material-1]). Characterization as MSCs was also confirmed for these cells through standard differentiation assays and colony forming unit analyses (unpublished results).

**Figure 1:**
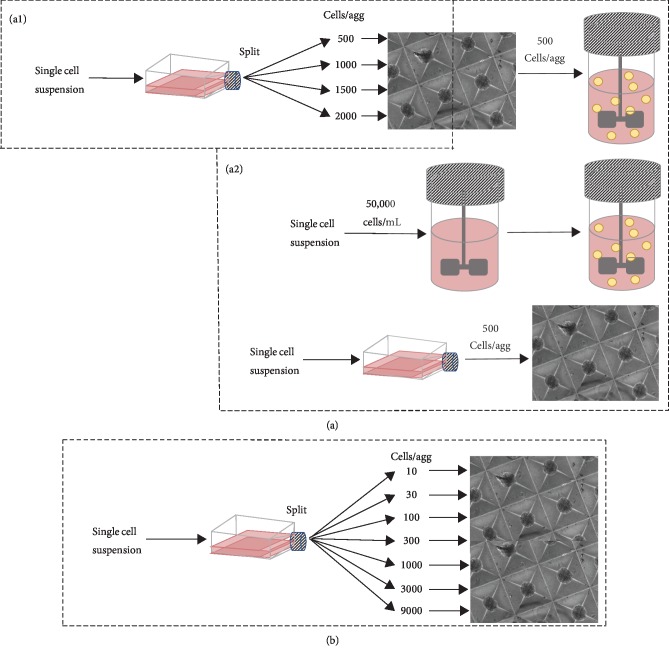
Overview of experiments. (a1) Microwell plates were seeded to form aggregates of varying cells/microwell to control the aggregate size that best matched the aggregates formed in bioreactors from single cells. (a2) Single cells and preformed aggregates were cultured in suspension bioreactors and compared to statically cultured aggregates. (b) Microwell plates were seeded to form aggregates of varying cells/microwell to investigate the dependence of macromolecule deposition on initial aggregate cell number.

**Figure 2:**
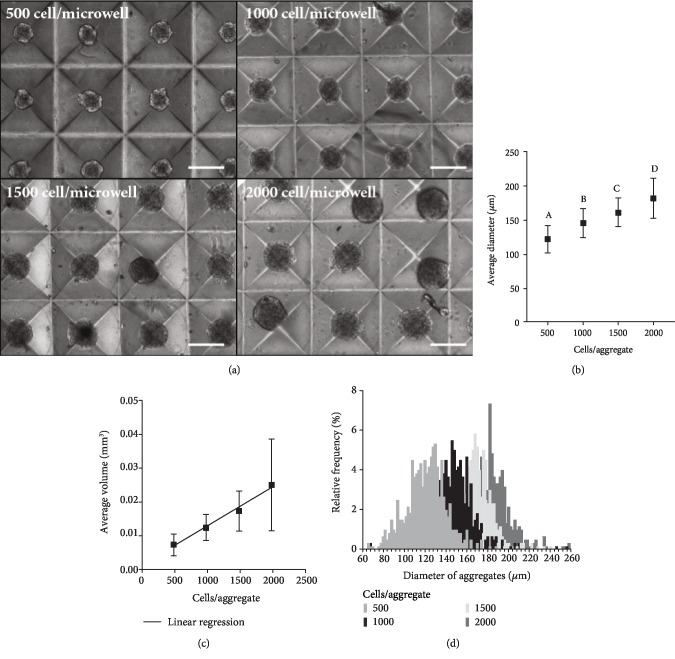
Analyses of microwell aggregates inoculated at 500, 1000, 1500, and 2000 cells/microwell after overnight formation. (a) Photomicrographs of aggregates in the microwells after overnight formation (scale bar = 200 *μ*m). (b) Average diameter of the aggregates formed in the microwell plates. Significance is denoted by alphabetical lettering; groups with no significance are linked by the same letters, while groups with significance do not share the same letters (*p* < 0.05). (c) Average volume of the aggregates formed in the microwell plates. Linear regression given by *y* = (1.161∗10^−5^) *x* + 1.084∗10^−3^ and *R*^2^ = 0.987. (d) Distribution of aggregate diameter based on the number of cells/aggregate. At each cell density, triplicate wells were inoculated and 100 aggregates from each well were individually sized in two perpendicular directions (*N* = 300).

Suspension bioreactors have been shown to be able to support stem cell population expansion and to also impact the characteristics of the resulting specialized cell populations derived from bioreactor expanded stem cells [12, 13, 25–30]. It was surmised that the outcome of a bioreactor-based cell expansion process would be affected by the form of the inoculum used. Specifically, the objective of this study was to compare the effect of inoculating bioreactors with (i) single cells which would go on to form aggregates within the vessel or (ii) inoculating a bioreactor with aggregates that had been preformed using microwell technology (Figure 1(a)). Aggregates of varying sizes can potentially have differences in cell-to-cell contact and nutrient diffusion, thereby affecting MSC viability and differentiation. As such, to ensure similar aggregate phenotypes between the two formation methods, the size of the aggregates formed in the microwell plates was investigated so that it in turn could be controlled to be similar in size to the aggregates formed from single cells within the suspension bioreactors [29]. Microwell plates were seeded to form aggregates of varying cells/microwell. Cells harvested from 2D flasks were inoculated into 24-well microwell plates in 0.8 mL at 500, 1000, 1500, and 2000 cells per aggregate, corresponding to between 750,000 and 300,000 cells/mL of medium, to determine the diameter distribution of the formed aggregates as a function of input cell numbers (Figure 1(a)). The cells condensed into aggregates overnight, and images of the well plates depicted the grades of aggregate size (Figure 2(a)). The average diameters (±standard deviation) of 121 ± 19.8, 145 ± 21.7, 161 ± 21.4, and 181 ± 29.4 *μ*m were generated by inoculating 500, 1000, 1500, and 2000 cells/microwell, respectively (Figures 2(b) and 2(d)). The aggregate volume (calculated using the measured diameters and assuming spherical geometry based on our previous observations [38]) was very well correlated (*R*^2^ = 0.987) with the number of cells inoculated per aggregate within the range of 500-2000 cells/aggregate (Figure 2(c)).

Since the use of the microwell plates allows for the overnight generation of aggregates with a tightly controlled size distribution, we hypothesized that once these aggregates were inoculated into the suspension bioreactors, it would result in a population of aggregates with a more uniform size distribution compared to the aggregates that result from inoculating single cells into a bioreactor. This is important because differing size can affect diffusion and cell-to-cell contact throughout the aggregate, which can, in turn, affect aggregate phenotype [17]. We therefore initially targeted the production of aggregates in microwells with sizes matching those generated by single cells in stirred suspension culture, with the hope that a longer period of stability at this size would result in greater cumulative deposition of extracellular matrix components.

In preliminary studies, single cells in bioreactors generated aggregates with an average diameter of 115.3 ± 14.6 *μ*m on culture day 8 and 137.1 ± 20.9 *μ*m on culture day 10 (Supplementary [Supplementary-material supplementary-material-1]). The closest average diameter in the static microwell after overnight formation was 121.1 ± 19.8 *μ*m (Figure 2(b)) when 500 cells were inoculated per microwell. We used 500 cells/aggregate to preform the aggregates used in suspension culture phase of the study (Figure 1(a)).

Stirred suspension bioreactors have a few advantages over the described microwell culture techniques including reduced manual intervention, the inherent ability to provide mechanical stimulation (shear), and if super aggregation is desired, it can be induced by decreasing the stir rate. We observed a greater frequency of unintended aggregate transfer between individual microwells during manual medium exchanges with the large aggregates than previously observed [54], potentially due to differences in size.

### 3.2. Cell Proliferation

**Figure 3:**
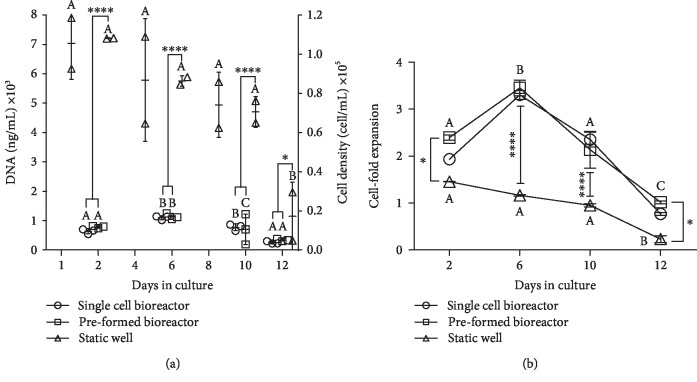
Cell proliferation in digested tissue-like aggregates as shown by (a) quantified DNA and (b) cell-fold expansion. DNA quantified using CyQUANT Cell Proliferation Assay Kit (Life Technologies). DNA in diploid primary cell assumed to be 6.6 pg/cell. Duplicate samples were harvested from the three bioreactors for the dynamic conditions and two wells from the static conditions, then subjected to quantification. Significance between culture time points (within conditions) is denoted by different alphabetical letters (*p* < 0.05). Significance between conditions is denoted by the presence of asterisks (^∗^*p* < 0.05, ^∗∗^*p* < 0.01, ^∗∗∗^*p* < 0.005, and ^∗∗∗∗^*p* < 0.001).

The aggregate formation approaches were evaluated to determine which specific protocol allowed for greater cell expansion from a bioprocessing prospective. For purposes of this comparison, time in culture since last passage was kept constant between the conditions. In the suspension bioreactor conditions, the inoculation density at culture day 0 was also kept constant at 50,000 cell/mL. DNA was quantified over the culture period as an indirect measurement of cell proliferation. Preformed aggregates were retained in the static microwell plates for the duration of the culture period for comparison of cell growth trends, aggregate size, and sGAG deposition between the static and dynamic culture systems. Levels of differentiation marker expression post-hMSC aggregation demonstrated enhanced chondrogenic expression (unpublished results). Similar DNA content was observed in the two suspension bioreactor conditions inoculated with either single cells or the preformed aggregates over the culture period (Figure 3). It also indicates that proliferation continued in both bioreactor conditions until they reached approximately a 3.5 cell-fold expansion, which occurred around culture day 6, at which point detectable proliferation ceased. The DNA content in the static well plates (seeded at 15-fold higher density) containing the preformed aggregates (approx. 1200 aggregates/well) showed a steady decrease in DNA content over the culture period (Figure 3).

This finding demonstrates that suspension bioreactors seeded at low density can support further proliferation of MSCs within aggregates, whereas aggregates seeded at much higher density in the static microwell plates have a more limited capacity in this regard. This is likely because the high initial seeding density was already at the upper limit of viability, although it is possible a role for shear experienced in the suspension bioreactor would be uncovered if compared with static cultures seeded at equivalent density.

### 3.3. Imaging and Sizing of Aggregates Cultured in Bioreactors

**Figure 4:**
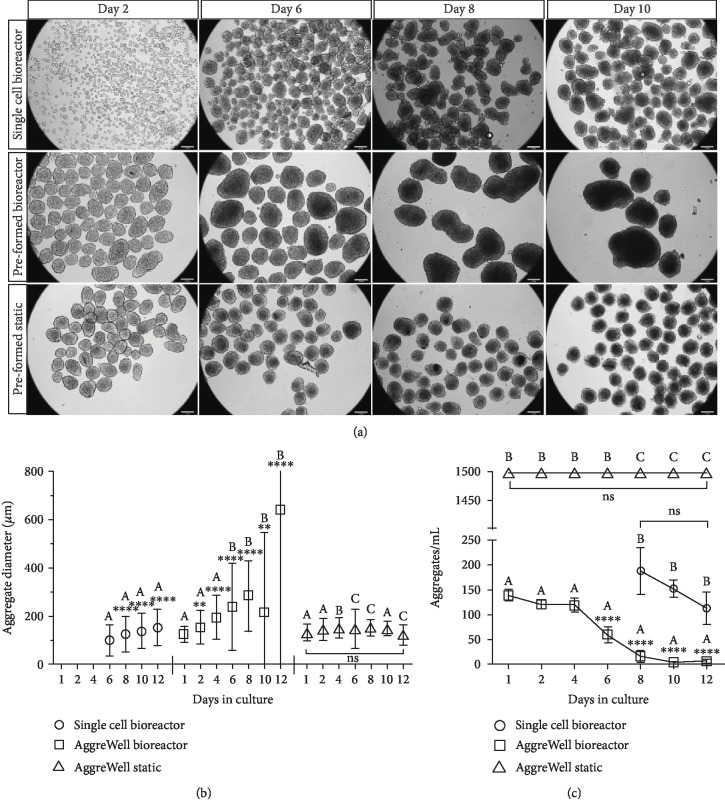
(a) Photomicrographs of aggregates harvested on day 2, day 6, day 8, and day 10 (scale bar = 140 *μ*m); (b) average aggregate diameter; and (c) aggregate density in the respective conditions over the culture period. From the suspension bioreactors, aggregates in 1 mL samples from three bioreactors were assessed. From the static culture, 10% of the aggregates from duplicate wells were assessed for diameter, and a value of 1200 aggregates per well (AggreWell 400, STEMCELL Technologies Inc.) was used for density analysis. Significance between conditions is denoted by different alphabetical letters (*p* < 0.05). Significance between day 1 and all future time points (within conditions) is denoted by the presence of asterisks (^∗^*p* < 0.05, ^∗∗^*p* < 0.01, ^∗∗∗^*p* < 0.005, and ^∗∗∗∗^*p* < 0.001).

After inoculation of single MSCs and preformed aggregates into the suspension bioreactors, samples of aggregates were harvested from the respective bioreactors for imaging and size assessment. The aggregates generated from single cells within the bioreactors started with a few single cells coming together. As such, sizing of the aggregates did not begin until day 6 when the aggregates were of a size that could be consistently measured (approximately 100 ± 60 *μ*m). At day 6, the average diameter was 100.8 ± 62.4 *μ*m, which increased over the remaining culture period to 125.0 ± 71.4 *μ*m on day 8, 137.5 ± 73.4 *μ*m on day 10, and 153.0 ± 75.4 *μ*m on day 12 (Figures 4(a) and 4(b)). Although there is some variation from preliminary experiments and the aggregates formed from single cells in the bioreactor are slightly larger than observed in preliminary studies (Supplementary [Supplementary-material supplementary-material-1]), the size between day 6 and day 8 still approximated the diameter of the static 500 cell/well condition (121 ± 19.8 *μ*m) (Figure 4(a)). As the cells did proliferate over the initial culture period, this growth was due to both aggregation and proliferation. The preformed aggregates that were inoculated into the bioreactors started at an average diameter of 124.9 ± 32.7 *μ*m on day 1 and increased to an average diameter of 643.5 ± 725.5 *μ*m by day 12. This aggregate size increase was not observed in the preformed aggregates that were kept in the individual microwells of the microwell plate. Therefore, the large size increase observed was most likely due to the agglomeration of aggregates into super aggregates within the bioreactor, but cell proliferation or production of ECM could have also contributed to this outcome.

Aggregate density was also determined for each culture condition (Figure 4(c)). In the single-cell bioreactor condition, the number of aggregates in a 1 mL sample was quantified from day 8 onwards as the high number of aggregates before this time point made manual microscopic counts inaccurate (Figure 4(a)). The density in this condition decreased from an average of 188 aggregates/mL on day 8 to 113 aggregates/mL on day 12. Whereas in the preformed bioreactor, the aggregate density decreased significantly more as the culture progressed from day 6 to day 12. This information, coupled with the significant increase in aggregate size over the culture period, suggests that many aggregates were fusing together into still larger structures within the bioreactor. This fusion or “super aggregation,” which has been shown to occur with other stem cell aggregates in static culture [55], also occurred in the bioreactors containing the initially smaller aggregates generated from single cells, but not to the same extent and with delayed kinetics.

According to previous studies, large aggregates beyond a critical diameter likely experience oxygen and nutrient mass transport limitations in their core [54, 56, 57]. Recently, it has been reported that hMSC aggregates show minimal reduction in oxygen tension, with only a 10-11% oxygen gradient with 50,000-60,000 cells per aggregate (approximately 300-350 *μ*m diameter) [52, 58]. It is important to note that low oxygen tension has been shown to upregulate chondrogenesis of MSCs [37, 59, 60]. However, mechanical stress due to cortical compaction, rather than oxygen depletion, has now been implicated to play a key role in metabolic reprograming of hMSCs [58]. In this study, the preformed bioreactor condition contained a population of aggregates that were highly variable in size wherein some aggregates exceeded 350 *μ*m diameter. As such, this condition may result in untoward differential metabolic reprograming of hMSCs. Consequently, future experiments are planned to assess the effects of seeding smaller preformed aggregates and the use of higher stir rates to mitigate super aggregation and control aggregate size.

### 3.4. sGAG Quantification

**Figure 5:**
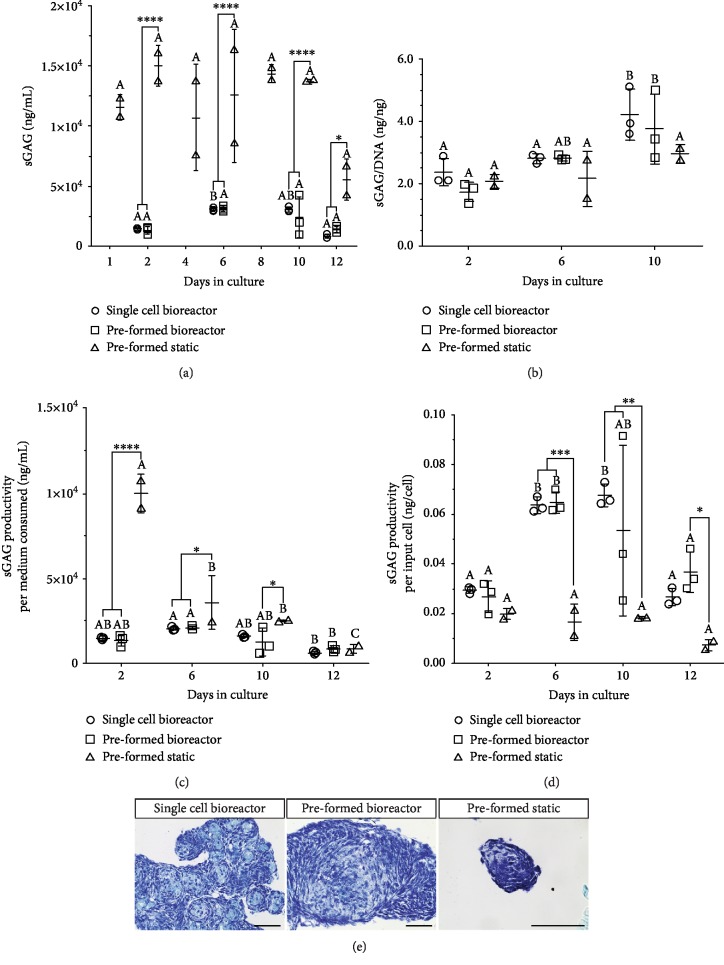
(a) sGAG and (b) sGAG/DNA quantification of digested tissue-like aggregates cultured within the suspension bioreactor conditions and the static well plates. (c) sGAG productivity per total consumed medium. (d) sGAG productivity per input cell number. sGAG quantified using DMMB reaction. DNA quantified using CyQUANT Cell Proliferation Assay Kit. Significance between culture time points (within conditions) is denoted by different alphabetical letters (*p* < 0.05). Significance between conditions is denoted by the presence of asterisks (^∗^*p* < 0.05, ^∗∗^*p* < 0.01, ^∗∗∗^*p* < 0.005, and ^∗∗∗∗^*p* < 0.001). (e) Toluidine blue staining of sGAG in day 12 aggregates from the suspension bioreactors inoculated with single cells, the suspension bioreactors inoculated with preformed aggregates, and the preformed aggregates kept in the static microwell plates. Scale bar = 100 *μ*m.

The sGAG levels retained within the aggregated MSCs followed a similar trend for both suspension bioreactor conditions (Figure 5(a)), increasing from day 2 to day 6 and then decreasing from day 10 to day 12. As cell death is evident later in the culture period (Figure 3), the decrease in sGAG may have been due to the loss of integrins and cadherins holding the cells/tissue together, resulting in the aggregates breaking apart and releasing ECM into the medium [37, 44, 45]. The sGAG content within the static well plate aggregates appeared to be higher but also to have a greater degree of variability between culture plates. As the data are depicted as sGAG per mL of culture medium, this finding could be due to the high density of cells within this condition.

The sGAG levels were normalized to DNA for each of the culture conditions on day 2, day 6, and day 10 (Figure 5(b)). Day 12 sGAG/DNA data was omitted due to the high degree of culture death at this time point (Figure 3). The highest sGAG/DNA ratios were observed at day 10 in single-cell bioreactor condition and preformed bioreactor condition, which was significantly greater than their respective condition at the start of the culture period (day 2). The highest sGAG/DNA observed was 4.22 ng/ng on average in the single-cell bioreactor condition on day 10 of culture. However, aggregates ranged between 0.5 and 6.5 ng/ng depending on the culture condition and length of culture. Variation in sGAG and sGAG/DNA values was seen to a degree in all culture conditions which could have been due to the variation in aggregate size. Previous literature reports that aggregate size can affect cell signaling, thereby affecting the amount of ECM deposited by the cells and resulting in a different tissue-like aggregate phenotype [37, 61].

sGAG productivity was also determined with the primary process-economy determinants being the number of input cells (sGAG per cell inoculated) and the total volume of culture medium consumed (sGAG per total mL of medium used) (Figures 5(c) and 5(d)). Cumulative medium consumption for the respective culture conditions is given in Supplementary [Supplementary-material supplementary-material-1]. Peak sGAG production per input cell reached a maximum for the single-cell condition on day 10 (0.068 ng/cell), for preformed aggregate on day 6 (0.065 ng/cell), and in the static cultures on day 2 (0.020 ng/cell). When expressed per mL of medium consumed, the peak values for the three conditions were 2042.1 ng/mL (day 6), 2067.2 ng/mL (day 6), and 11,547.1 ng/mL (day 1). Given these results, bioreactor culture day 6 proves to be an ideal time point for tissue engineering applications or changing the culture parameters to overcome the subsequent drop in productivity. The sGAG productivity relative to medium consumed being highest at day 1 in the preformed static condition is a reflection of the small volume of medium used in the static well plates relative to cell density.

A normal articular cartilage sample is expected to contain 83-250 (ng/ng) sGAG/DNA [43]; however, this represents the total accumulated within the cartilage matrix over a long period of development. Peak sGAG/DNA levels of ~4 ng/ng attained in this study were slightly higher than those in a previous report induced in 20% oxygen tension within a chondrogenic medium (~2 ng/ng), but slightly lower than those subjected to 2% oxygen tension (~5 ng/ng) [37]. In another study, static cell pellets in a chondrogenic medium containing TGF-*β*1 and dexamethasone were shown to contain 2 (ng/ng) sGAG/DNA at culture day 6 but contained more than 10 (ng/ng) sGAG/DNA when the culture was extended to 14 days [44]. The medium used in the present study (PPRF-msc6) is formulated for the growth of MSCs yet does contain small amounts of TGF-*β*1. It would be interesting for future studies to uncover the effects of lower oxygen tension, chondrogenic medium, and a longer culture period within the suspension bioreactor system.

### 3.5. ECM Visualization and Quantification

Although quantification analysis gives some insight into the production of ECM macromolecules, it is also important to visualize this production to determine the distribution and possible identity of ECM components. The aggregates in each culture condition were harvested for histological analysis of collagen type I, collagen type II, and sGAG after 12 days of culture. No visible staining of sGAG, as depicted by metachromatic (light purple) staining, was evident throughout the aggregates for any of the culture conditions (Figure 5(e)). Toluidine blue also has high affinity to DNA, which is demonstrated by dark blue staining. The high density of cells (dark blue) in the aggregates is consistent with the large amounts of DNA quantified over the culture period, and the apparent lack of staining for sGAG is consistent with the relatively low amount of sGAG quantified in comparison with native tissues. In all conditions, but most pronounced for the aggregates formed from single cells in bioreactors, total ECM accumulation between the cells was also evident with this stain.

**Figure 6:**
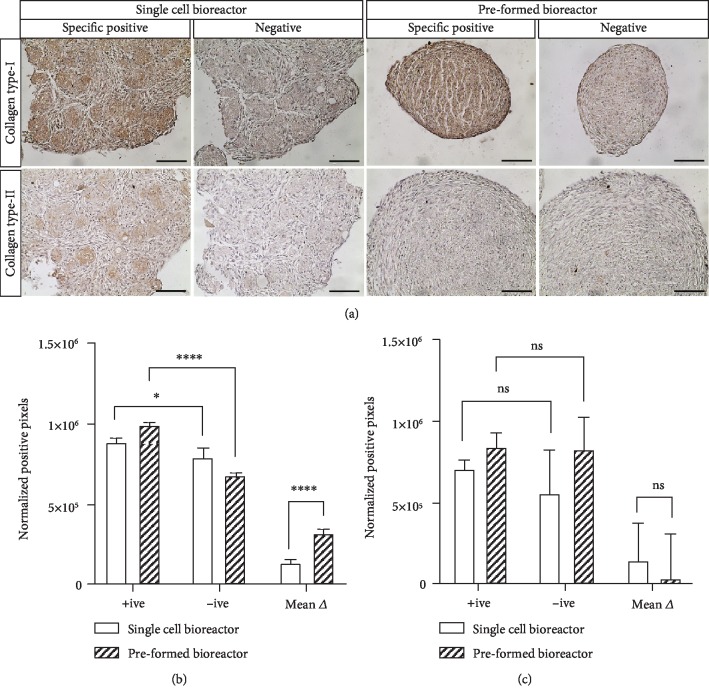
IHC of collagen deposition in aggregates harvested on culture day 12. Sections were either incubated with the primary antibody (positive) or had the primary antibody omitted (negative). (a) IHC staining of collagen type I and collagen type II in the aggregates formed within the suspension bioreactors. (b) Collagen I and (c) collagen II quantification depicted as normalized positive staining (image pixels) in sections incubated with the primary antibody (positive), had the primary antibody omitted (negative), and the mean difference (mean Δ) between these two. DAB signal intensity quantified in IHC images by ImageJ and an open source IHC quantification plugin [46]. Significance differences are denoted by the presence of asterisks (^∗^*p* < 0.05, ^∗∗∗∗^*p* < 0.001).

Collagen type I staining was visually evident in the aggregates from both the preformed single-cell bioreactor conditions, whereas collagen type II was less, albeit still visually evident in the single-cell condition (Figure 6(a)). Collagen type I and type II staining in the aggregates from the single-cell bioreactor condition was primarily localized in pockets between the cells. Collagen type I within the preformed aggregates appeared to be located throughout the aggregate. The exact reason for this difference in architecture is undetermined; however, we hypothesize that it may be due to differences in how the aggregates were formed. When the aggregates are preformed and then inoculated into the bioreactor, the entirety of the aggregate has hypothetically equal opportunity to deposit ECM, resulting in a more even distribution. When single cells are inoculated into the bioreactor, some cells may come together to form an aggregate before others, and smaller aggregates may further conglomerate over the culture period. This more complex history may result in ECM distributed in pockets throughout the aggregate.

Quantification of collagen type I (Figure 6(b)) shows significant positive staining in the sections incubated with the primary antibody (positive) as compared to sections that had the primary antibody omitted (negative) for both bioreactor conditions. The mean difference (mean Δ) between the positive and negative sections shows significantly more collagen type I staining in the preformed bioreactor aggregates as compared to the single-cell aggregates. IHC quantification for collagen type II (Figure 6(c)) did not show significant positive staining between the sections incubated with the primary antibody (positive) and those that had the primary antibody omitted (negative). As such, we cannot conclude that there was any significant collagen type II expression in either of the bioreactor conditions.

**Figure 7:**
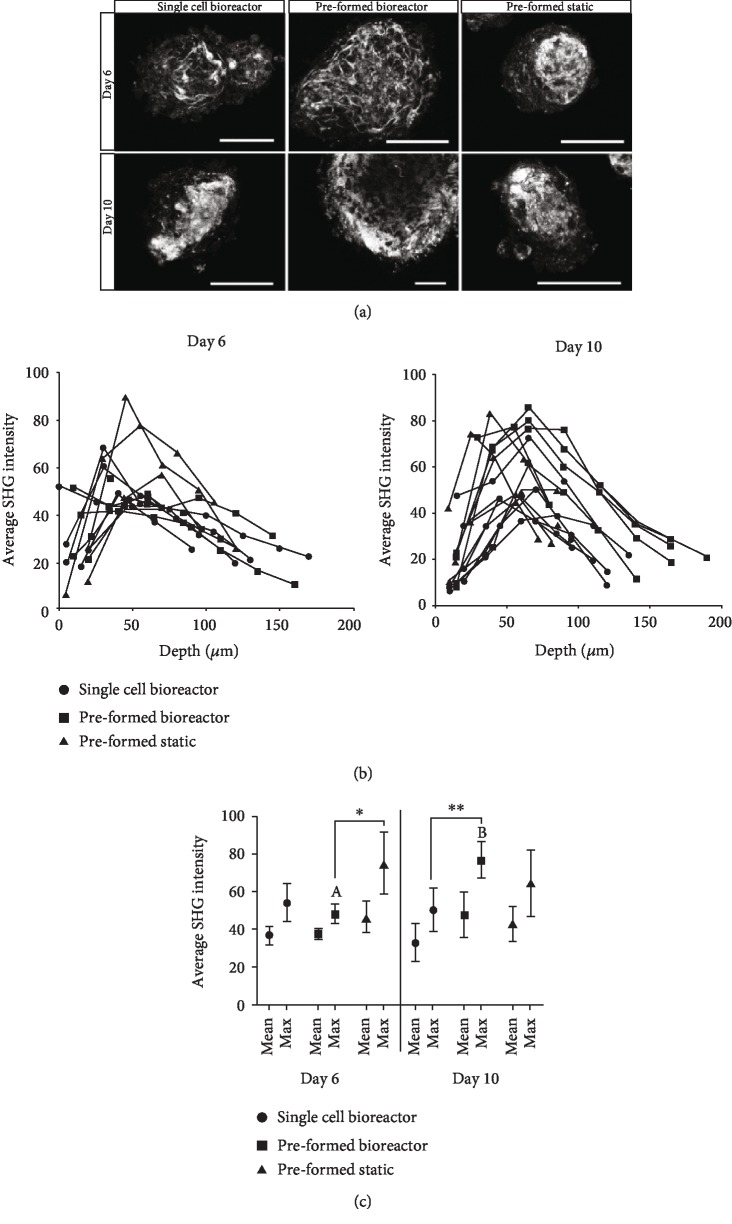
SHG of collagen deposition in aggregates harvested on culture day 12. (a) SHG images of collagen fibrils in the 3D aggregates. Scale bar = 100 *μ*m. (b) SHG signal intensity throughout the depth of the aggregates. (c) Mean and maximum SHG intensity at the respective time points and culture conditions. SHG signal intensity quantified by ImageJ. Significance between culture time points (within conditions) is denoted by different alphabetical letters (*p* < 0.05); absence of lettering meaning no significant difference. Significance between conditions is denoted by the presence of asterisks (^∗^*p* < 0.05, ^∗∗^*p* < 0.01).

Second harmonic generation (SHG) is a noninvasive imaging method to visualize collagen production in 3D tissue constructs [47] and thus can confirm the IHC staining results. Although SHG is highly sensitive for collagen I and II fibrils, it is unable to distinguish between collagen types [62]. At culture days 6 and 8, the aggregates from the single-cell bioreactor condition had distinct pockets of intense collagen emission signal with decreased intensity in the regions surrounding these pockets (Figure 7(a)). These findings parallel the IHC staining. In the aggregates from preformed bioreactors, the collagen was distributed more evenly, especially on the cortex of the aggregate. In the preformed static aggregates, collagen was either located in a central pocket or more evenly distributed throughout the small aggregates. When the SHG signal was quantified using ImageJ, the majority of the aggregates in all conditions had maximum signal located in the middle of the aggregate (Figure 7(b)). The mean SHG intensity of the aggregates in a condition, summed over all the sections, was the highest for the preformed bioreactor aggregates at day 10 (48.0 ± 11.9) and the preformed static aggregates at day 6 (46.9 ± 8.0) (Figure 7(c)). The aggregate slice with the maximum SHG (collagen) signal was averaged for all the aggregates in a condition, and again, the values were the highest for the preformed bioreactor aggregates at day 10 (76.7 ± 9.0) and the preformed static aggregates at day 6 (75.1 ± 16.4). These findings parallel the IHC quantification results.

Throughout both of the suspension conditions, yet most pronounced in the preformed bioreactors, collagen staining at the core of the aggregates was inversely related to aggregate size (Figure 7(b)). This phenomenon was also observed by Markway et al. ([37], see Figures 3 and 4) and confirms that the preformed aggregates employed here were likely too large for efficient chondrogenesis. As Markway and colleagues posited, the amount of oxygen and nutrients the cells received through diffusion processes could have been limited, which in turn could have affected the amount of ECM the cells produced within the aggregates. Within our experiments, the cells in the outer layers of the aggregate could be responding to mechanical forces, in this case shear, which is known to upregulate ECM production in MSCs [34, 45, 63, 64]. We therefore propose that a bioreactor system which enables the formation of smaller aggregates, either from single cells or possibly preformed aggregates at higher impellor rates, be used in future experiments investigating the role of low-oxygen tension and chondrogenic growth medium.

### 3.6. Dependence of Macromolecule Deposition on Initial Aggregate Cell Number

As shown above, there were clear differences between smaller aggregates formed in stirred suspension from single cells and substantially larger preformed aggregates inoculated and cultured in suspension bioreactors. We therefore took advantage of the ability of microwell-forced aggregation to precisely control aggregate size to generate a range of sizes, which were maintained within the static microwells in which they were formed for 8 days and then harvested for analysis.

**Figure 8:**
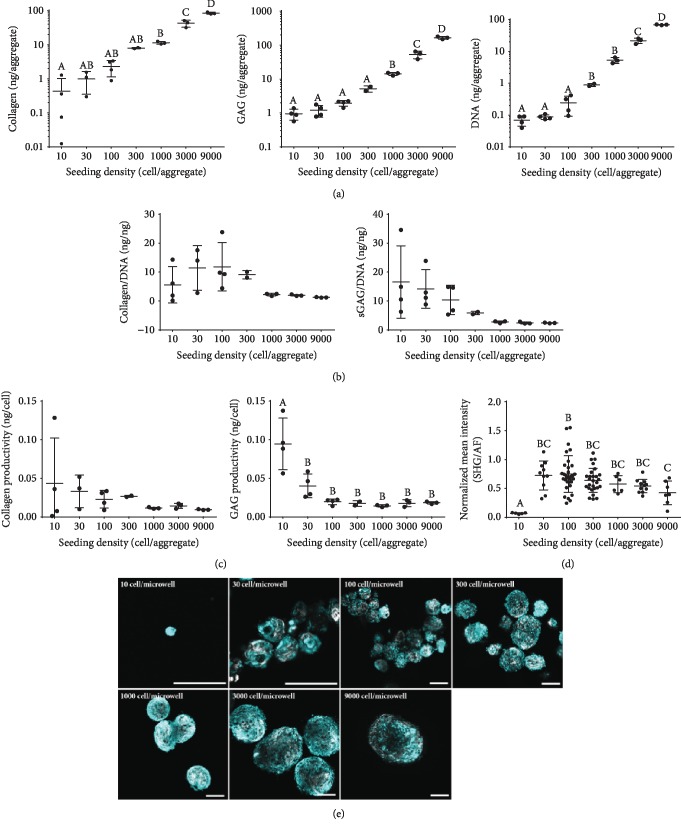
Analysis of static aggregates of different sizes cultured for 8 days. Collagen and sGAG quantification in digested tissue-like aggregates shown by (a) total weight, (b) normalized to the DNA content, and (c) productivity per input cell number. Collagen quantified using hydroxyproline quantification. sGAG quantified using DMMB reaction. DNA quantified using CyQUANT Cell Proliferation Assay Kit. Significance between aggregate sizes is denoted by different alphabetical letters (*p* < 0.05); absence of lettering on graph meaning no significant difference. Collagen fibrils imaged using SHG (grey). All other ECM and cells were imaged using AF (teal). Intensity of signal quantified using ImageJ. (c) SHG intensity normalized to AF intensity. (d) SHG images overlaid with AF images. Scale bar = 100 *μ*m.

As expected, the DNA quantified per aggregate was positively correlated with the increasing number of cells originally inoculated in each aggregate, even after 8 days of culture (Figure 8(a)). Additionally, the collagen and sGAG quantified per aggregate also increased with the number of cells in each aggregate. When the collagen and sGAG were normalized to the DNA, the trends differed (Figure 8(b)). Higher amounts of collagen/DNA were observed in smaller aggregates ranging from 10 to 300 cells/aggregate, as compared to larger aggregates ranging from 1000 to 9000 cells/aggregate. Similarly, higher sGAG/DNA ratios were detected in smaller aggregates ranging from 10 to 30 cells/aggregate, with a decreasing trend of aggregates larger than 100 cells/aggregate. However, there were larger variations of both sGAG and collagen in the smaller aggregates, and significant differences were not observed.

Also determined was sGAG and collagen productivity with the primary process-economy determinant being the number of input cells (sGAG per cell inoculated) (Figure 8(c)). Productivity for collagen was the highest at 10, 30, 100, and 300 cells/aggregate (0.043, 0.033, 0.023, and 0.027 ng/cell, respectively) yet highly variable when the aggregate contained less than 100 cells. As such, significant differences were not observed. Productivity for sGAG was the highest at 10 and 30 cells/aggregate (0.095 and 0.040 ng/cell) yet again highly variable at these lower seeding densities. Again, significant differences were not observed. SHG and AF imaging was used to visualize the collagen production and organization in the aggregates of different sizes (Figure 8(e)), and the signal intensities were quantified using ImageJ (Figure 8(d)). Both visually and when quantified, the collagen production was the highest in the aggregates containing 30-1000 cells. Furthermore, the collagen in these sizes appears to be evenly distributed throughout the aggregate. In the aggregates with 3000 cells/aggregate, there starts to be a lack of collagen staining on the inside of some of the aggregates. With 9000 cells/aggregate, the lack of collagen staining on the inside of the aggregates is quite evident, with most collagen located on the periphery. This demonstrates that macromolecule deposition within the aggregates is modulated by aggregate size.

While very small aggregates can achieve a higher amount of sGAG/DNA or collagen/DNA on average, there is a much larger variability, likely reflecting the increased impact of individual cell gain/loss events at this scale. As such, aggregates containing between 100 and 300 cells may be optimal from both a productivity standpoint and a bioprocessing standpoint with regard to aggregate uniformity and nutrition diffusion within the aggregate.

Previous research has been conducted to investigate the effect of stem cell aggregate size on the phenotype of the resulting aggregate tissue [11, 36, 37]. Here, we add to this understanding by assessing a wide range of sizes of MSC aggregates specifically and show trends that can guide future research questions in this area. As the end goal within our lab is to produce tissue in a scalable and reproducible manner that is primed to differentiate towards a cartilage lineage, these results are informative for suspension bioreactor culture studies, where aggregate size can be manipulated by controlling shear.

## 4. Conclusions

This study has demonstrated that 3D tissue-like structures can be successfully produced in microwell plates and scalable suspension bioreactors under serum-free conditions using MSCs as a cell source and has characterized the resulting aggregates. Cells in bioreactor-generated aggregates maintained their capacity to produce ECM, representing a progression in the knowledge regarding scalable options for the production of MSC-based tissue for cartilage repair applications. When single-cell and 500-cell preformed aggregates were inoculated into suspension bioreactors, they both achieved similar cell-fold expansion and sGAG productivity measures. The 500-cell preformed aggregates showed enhanced collagen production throughout the entirety of the aggregate; however, ongoing super aggregation and variability in aggregate diameter diminished this result. When aggregates containing 10-9000 cells/aggregate were statically cultured, peak sGAG/DNA and sGAG productivity was obtained in 10-30 cells/aggregate; however, 100-300 cells/aggregate is almost as good and has significantly less variability. The combination of detrimental effects in excessively large aggregates with ongoing super aggregation indicates that bioreactor inoculation with single cells, smaller preformed aggregates, and/or manipulations to prevent super aggregation may yield improved outcome. Future work should be directed towards assessing the interacting effects of reduced oxygen tension, aggregate size, and chondrogenic induction medium on MSC aggregates within stirred suspension and static environments.

## Data Availability

The laboratory data used to support the findings of this study are included within the article or within the supplementary information file(s).

## Supplementary Materials

Supplementary Materials Figure S1: (a) distribution of aggregate diameter and (b) average aggregate diameter in replicate bioreactors over the 16-day culture. Suspension bioreactors were inoculated at day 0 with 50,000 MSCs/mL in serum-free medium (PPRF-msc6). Aggregates in 1 mL samples were assessed every two days after aggregate formation. Figure S2: GAG standard curve generated using known concentrations of chondroitin sulphate and the DMMB reaction. Figure S3: morphology of human SF-MSCs at different time points, as they are being expanded under serum-free conditions. Scale bar = 200 *μ*m. Table S1: antibodies and respective surface marker expression of the hMSCs used in this work (donor A). Figure S4: cumulative medium consumption (mL) in the suspension bioreactors (both inoculated with single cells and preformed aggregates) and static well plates over the 12-day culture period. Suspension bioreactors were inoculated with 125 mL of serum-free medium containing 6.25 million MSCs. Static well plates were inoculated with 0.8 mL of serum-free medium containing 600,000 MSCs.
