# Supplementary material for: Serum-Free Culture of Human Mesenchymal Stem Cell Aggregates in Suspension Bioreactors for Tissue Engineering Applications
Source: Stem Cells Int. 2019 Nov 7;2019:4607461. doi: 10.1155/2019/4607461 (PMC6878794; doi:10.1155/2019/4607461)
Supplement: Supplementary Materials — Figure S1: (a) distribution of aggregate diameter and (b) average aggregate diameter in replicate bioreactors over the 16-day culture. Suspension bioreactors were inoculated at day 0 with 50,000 MSCs/mL in serum-free medium (PPRF-msc6). Aggregates in 1 mL samples were assessed every two days after aggregate formation. Figure S2: GAG standard curve generated using known concentrations of chondroitin sulphate and the DMMB reaction. Figure S3: morphology of human SF-MSCs at different time points, as they are being expanded under serum-free conditions. Scale bar = 200 μm. Table S1: antibodies and respective surface marker expression of the hMSCs used in this work (donor A). Figure S4: cumulative medium consumption (mL) in the suspension bioreactors (both inoculated with single cells and preformed aggregates) and static well plates over the 12-day culture period. Suspension bioreactors were inoculated with 125 mL of serum-free medium containing 6.25 million MSCs. Static well plates were inoculated with 0.8 mL of serum-free medium containing 600,000 MSCs. [file 4607461.f1.docx]

## Supplementary Material


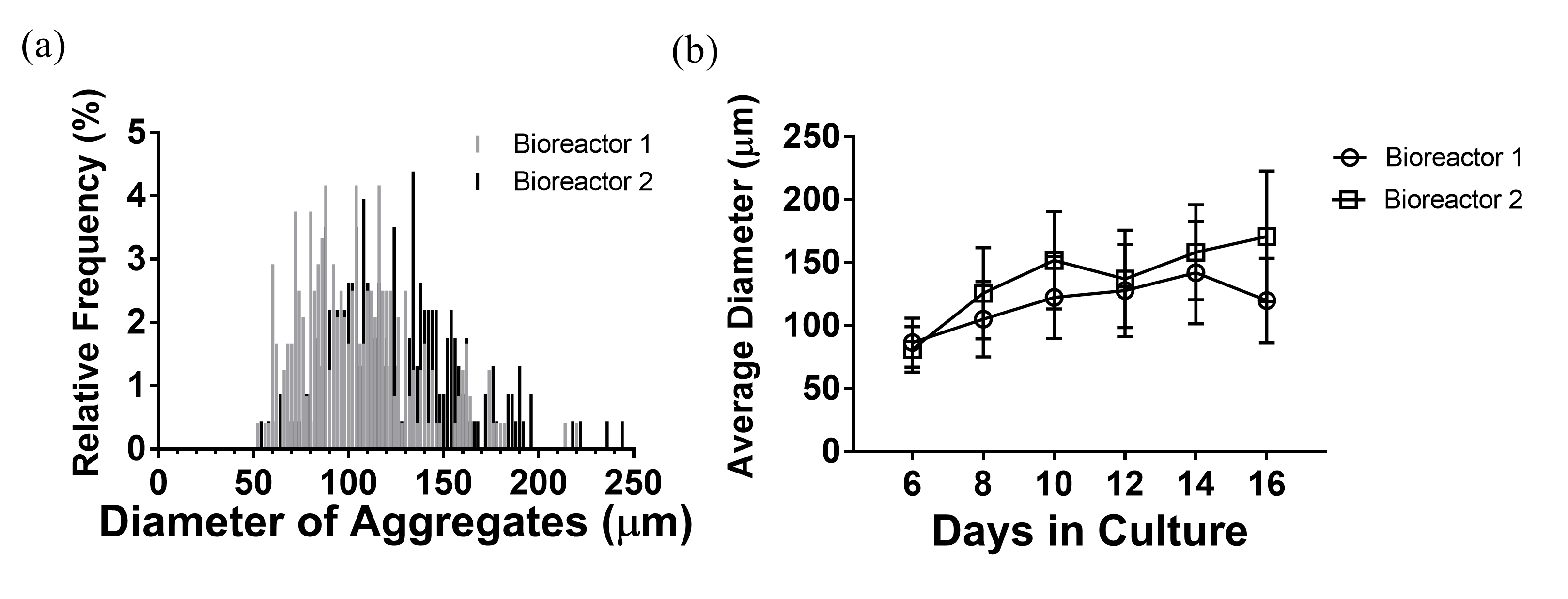
Figure S1. (a) Distribution of aggregate diameter and (b) average aggregate diameter in replicate bioreactors over the 16-day culture. Suspension bioreactors were inoculated at day 0 with 50,000 MSCs/mL in serum-free medium (PPRF-msc6). Aggregates in 1 mL samples were assessed every two days after aggregate formation.


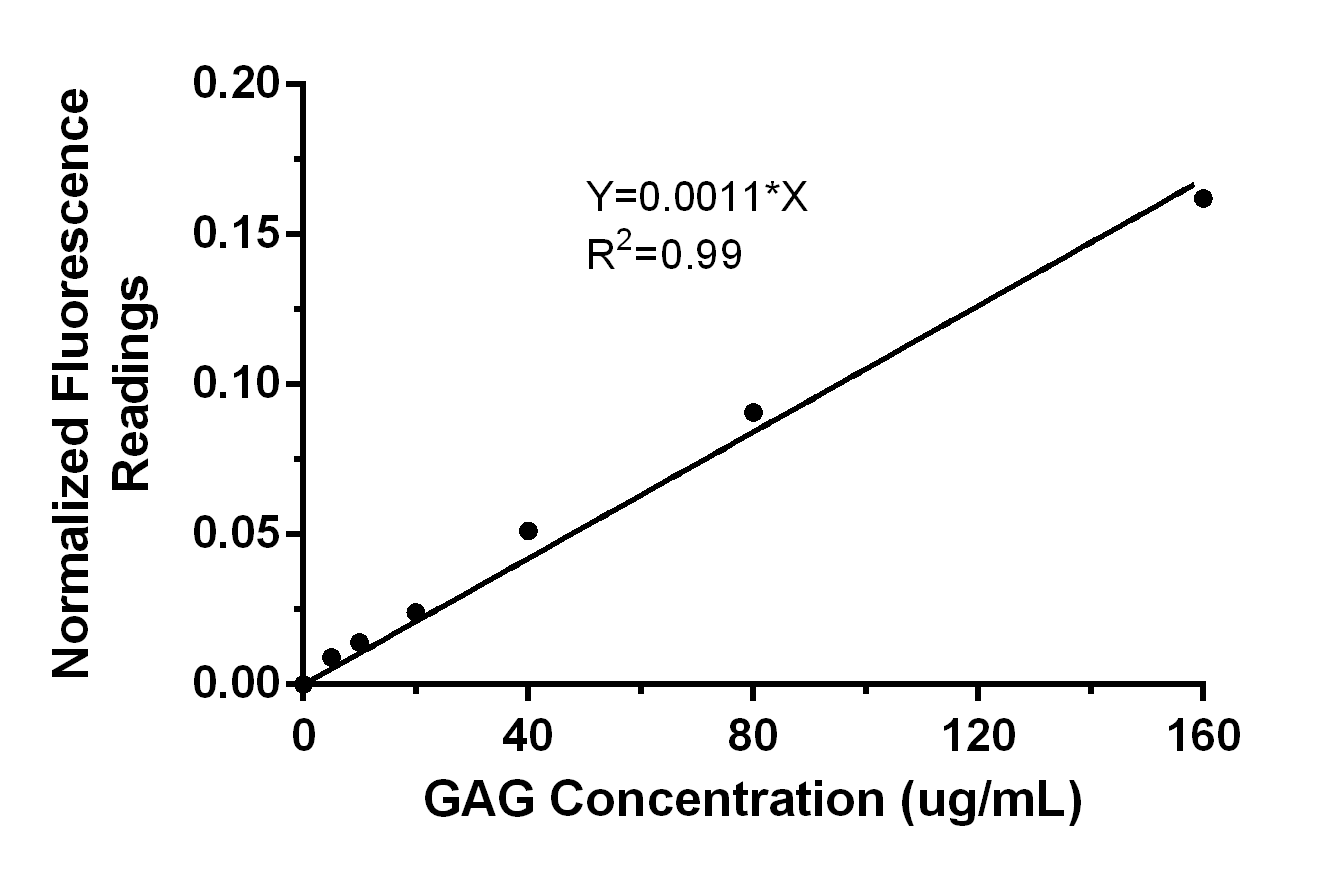


Figure S2. GAG standard curve generated using known concentrations of chondroitin sulphate and the DMMB reaction.


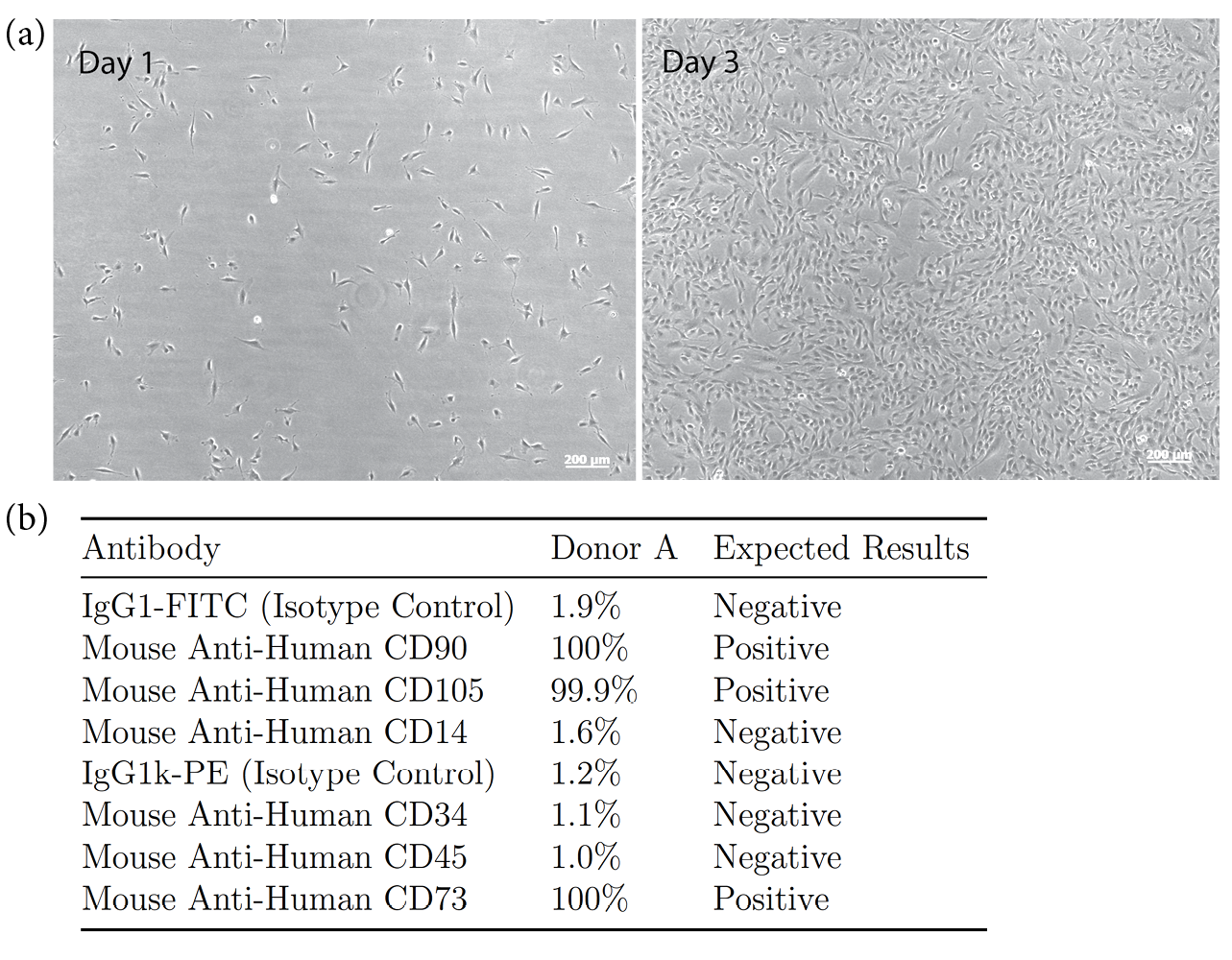


Figure S3. Morphology of human SF-MSCs at different time-points, as they are being expanded under serum-free conditions. Scale bar = 200 μm.

Table S1. Antibodies and respective surface marker expression of the hMSCs used in this work (Donor A).


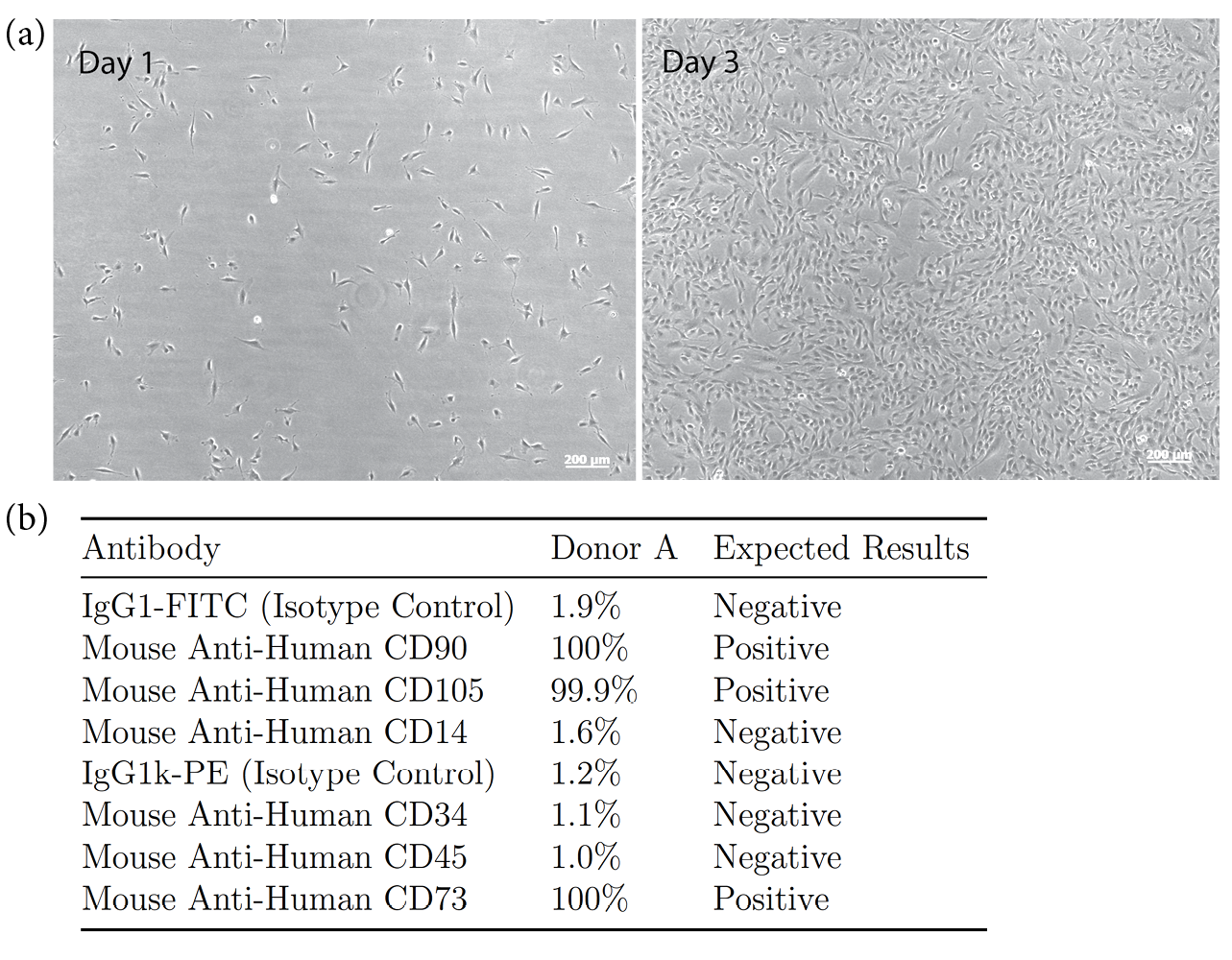


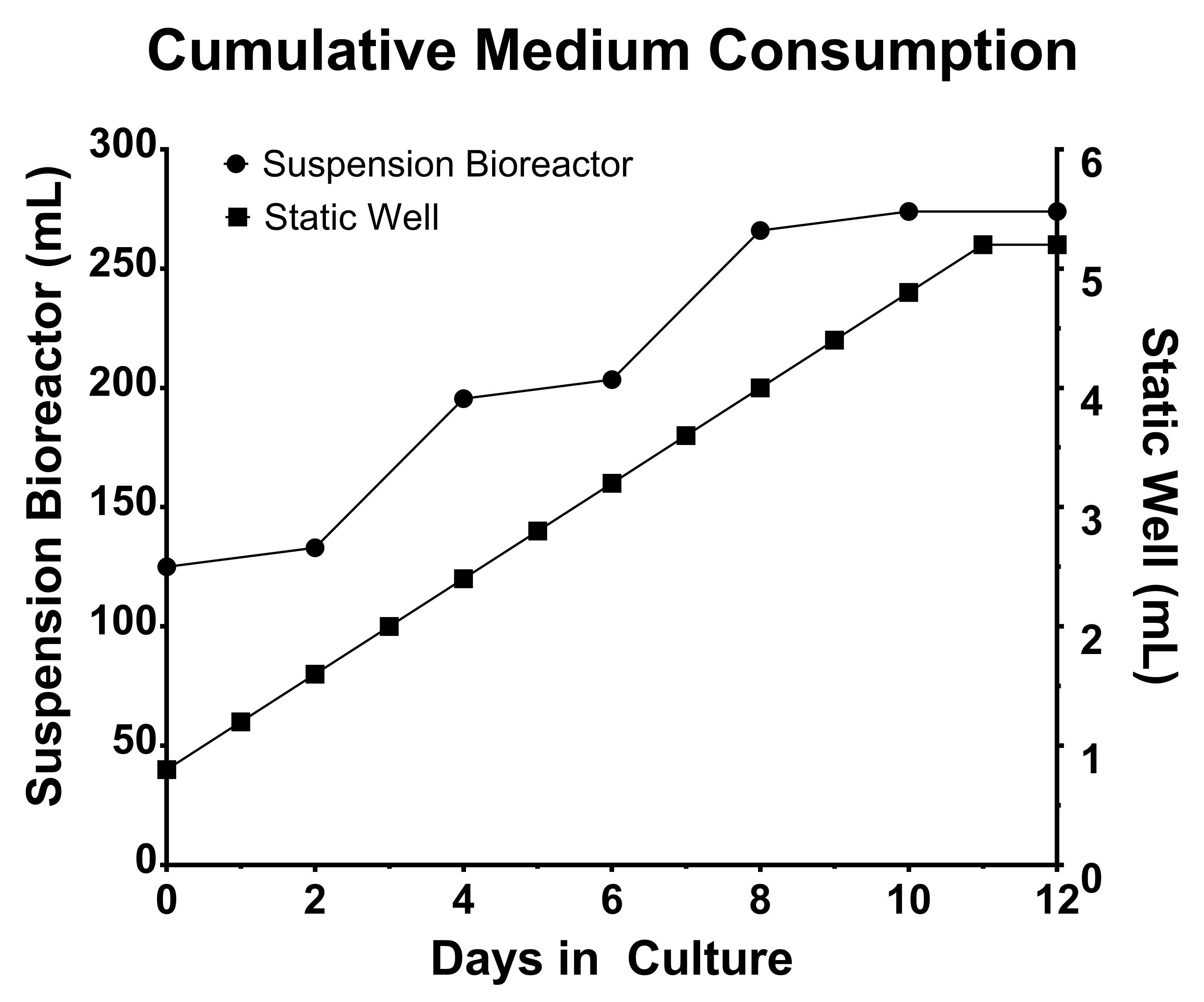


Figure S4. Cumulative medium consumption (mL) in the suspension bioreactors (both inoculated with single cells and pre-formed aggregates) and static well plates over the 12-day culture period. Suspension bioreactors were inoculated with 125 mL of serum-free medium containing 6.25 million MSCs. Static well plates were inoculated with 0.8 mL of serum-free medium containing 600,000 MSCs.
